# Recurrent Intra-abdominal Low-Grade Fibromyxoid Sarcoma Managed With Multivisceral Resections: A Case Report

**DOI:** 10.7759/cureus.107100

**Published:** 2026-04-15

**Authors:** Kiril Kirov, Presian Varbanov, Nikolay Mirinchev

**Affiliations:** 1 Department of Surgery, Faculty of Medicine, Burgas State University “Prof. Dr. Assen Zlatarov”, Burgas, BGR; 2 Department of Surgical Oncology, Complex Oncology Center Burgas, Burgas, BGR; 3 Department of Urology, Faculty of Medicine, Burgas State University “Prof. Dr. Assen Zlatarov”, Burgas, BGR

**Keywords:** intra-abdominal sarcoma, low-grade fibromyxoid sarcoma, muc4, multivisceral resection, recurrence, surgical oncology

## Abstract

Low-grade fibromyxoid sarcoma (LGFMS) is a rare soft-tissue sarcoma with deceptively bland histology. There is a recognized risk of late local recurrence and metastasis. Intra-abdominal presentations are uncommon and may remain clinically silent until the tumor is large, at which point complete resection can require multivisceral surgery. We report a 61-year-old woman diagnosed with intra-abdominal LGFMS in 2021. Histopathology showed classic fibromyxoid architecture with strong MUC4 expression, focal CD34 positivity, and a Ki-67 index of approximately 6%. After a margin-negative ileocecal resection, systemic therapy was administered. Multifocal intra-abdominal recurrence developed in early 2024 and was completely resected. Further recurrence occurred in 2025. The tumor involved the sigmoid colon, small bowel, urinary bladder, and spleen. Wide multivisceral resection was performed, including sigmoid colectomy, two small-bowel resections, partial cystectomy, splenectomy, and excision of additional nodules. Pathology confirmed recurrent LGFMS consistent with the original tumor, with negative surgical margins. The patient remains disease-free six months after the most recent surgery. This case underscores the propensity for persistent intra-abdominal LGFMS recurrence and supports complete surgical excision, often requiring multivisceral resection, as a key strategy for disease control in resectable recurrent disease.

## Introduction

Low-grade fibromyxoid sarcoma (LGFMS) is a rare fibroblastic neoplasm that can appear histologically bland while retaining the capacity for local recurrence and late metastatic spread. LGFMS was originally characterized as a metastasizing tumor with deceptively benign morphology, a paradox that remains central to its clinical behavior [1]. Subsequent clinicopathologic and molecular summaries have reinforced its prolonged and sometimes unpredictable course, including relapse many years after apparently complete excision [2].

Most LGFMS arise in the deep soft tissues of the extremities or trunk. Intra-abdominal and peritoneal presentations are less common and may remain clinically silent until tumors reach a large size, when complete resection can be technically demanding [2]. Late metastases can occur, most commonly to the lungs. Rare metastases to other sites, including the prostate and liver, have also been reported [2]. Histologic diagnosis is based on alternating fibrous and myxoid zones composed of uniform spindle cells with low mitotic activity, and immunohistochemistry is often pivotal when molecular testing is unavailable. In this setting, MUC4 expression is a highly sensitive marker that helps distinguish LGFMS from several morphologic mimics [3].

When available, molecular confirmation strengthens diagnostic confidence. LGFMS is strongly associated with FUS/CREB3L2 rearrangement, with less frequent variant fusions such as FUS/CREB3L1 [4,5]. LGFMS also overlaps morphologically with hyalinizing spindle cell tumor with giant rosettes and may sit on a spectrum with sclerosing epithelioid fibrosarcoma (SEF), emphasizing the importance of clinicopathologic correlation and appropriate ancillary testing [6,7].

Therapeutically, LGFMS is primarily a surgical disease. In a population-based series, durable disease control was most often observed with repeated selective surgery of operable metastases, whereas chemotherapy responses were limited [8]. Contemporary sarcoma guidelines emphasize complete resection with negative margins as the cornerstone of potentially curative treatment for resectable soft-tissue sarcomas, while acknowledging the limited role of radiotherapy in selected situations and the variable benefit of systemic therapy depending on histology and clinical context [9]. In localized soft-tissue sarcoma, perioperative anthracycline-ifosfamide chemotherapy may be considered selectively in patients at high risk of death rather than used routinely after complete resection [9]. When systemic therapy is used for advanced or unresectable soft-tissue sarcoma, doxorubicin-based chemotherapy remains the most common first-line approach, either as single-agent doxorubicin or in combination with ifosfamide in selected patients [9]. Other agents and combinations may be considered in later lines based on histology and prior therapy [9]. Available outcome data in advanced LGFMS/SEF suggest limited and inconsistent benefit from systemic regimens, with disease stabilization more common than durable tumor control, reinforcing the centrality of surgery whenever complete resection is feasible [10,11]. We present a case of recurrent intra-abdominal LGFMS managed surgically over four years, culminating in planned multivisceral resection for multifocal involvement.

## Case presentation

A 61-year-old woman first presented in 2021 with progressive abdominal heaviness and distension. Appetite was preserved, and she denied unintentional weight loss. Abdominal ultrasound identified a large intra-abdominal mass occupying most of the lower abdomen. In October 2021, she was admitted to our surgical oncology unit. Preoperative imaging findings are summarized from the available contrast-enhanced CT report, which described a large peritoneal/mesenteric mass and abdominal lymphadenopathy. The original CT images from 2021 were not available for review or inclusion, and nodal malignancy was not histologically confirmed.

At laparotomy, a 21 x 15 cm tumor arising from the mesentery with involvement of the terminal ileum was identified. An ileocecal resection with stapled anastomosis was performed, with negative margins.

Histopathologic examination supported the diagnosis of LGFMS based on characteristic morphology and immunohistochemistry, including strong MUC4 expression with focal CD34 positivity and a Ki-67 index of approximately 6% (SMA and DOG1 negative). Molecular testing was not available. Postoperatively, systemic therapy was administered at an external oncology center where the patient was followed. Details regarding the indication and regimen were not available in the records accessible to our surgical team. At discharge, we recommended surveillance with contrast-enhanced CT every six months.

In early 2024, surveillance imaging raised concern for recurrence. At laparotomy, a 7 x 6 cm tumor mass was identified lateral to the sigmoid colon. Adhesiolysis was performed, and the tumor was mobilized and completely excised using a no-touch technique to avoid tumor violation and spillage. No macroscopic local or distant metastases were observed intraoperatively. Surgical margins were negative, and pathology confirmed recurrent LGFMS. Postoperatively, systemic therapy was administered at the patient’s follow-up oncology center, but regimen details were not documented in the records available to our surgical team.

**Figure 1:**
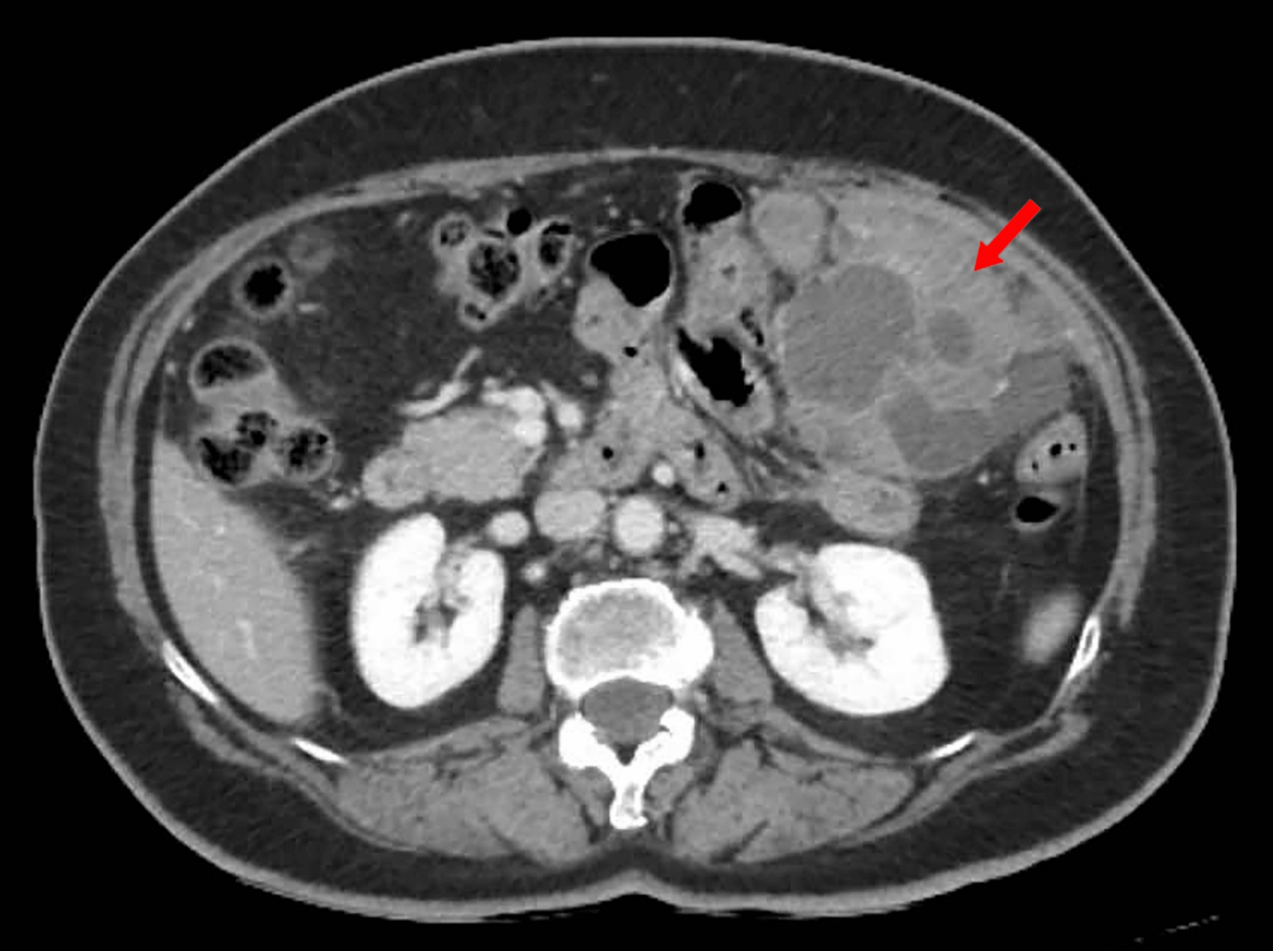
Contrast-enhanced CT demonstrating recurrent intra-abdominal tumor in 2025 Representative axial contrast-enhanced CT image shows a large intra-abdominal mass (arrow) corresponding to the lesion resected during the July 2025 multivisceral operation.

**Figure 2:**
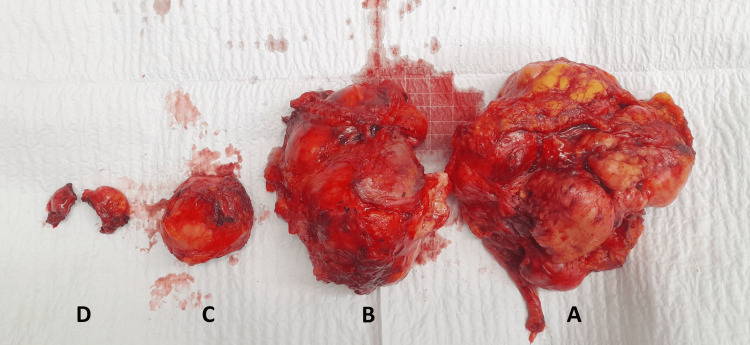
Gross surgical specimens from the 2025 multivisceral resection for recurrent intra-abdominal low-grade fibromyxoid sarcoma (A) Left lower quadrant mass resected en bloc with the involved sigmoid colon, urinary bladder, and two small-bowel loops. (B) Left upper quadrant mass removed en bloc with splenectomy. (C) Abdominal wall lesion resected with the involved rectus abdominis muscle. (D) Additional excised nodules.

In July 2025, the patient was admitted for recurrent disease. Preoperative contrast-enhanced CT demonstrated recurrent intra-abdominal disease (Figure 1). At laparotomy, dense adhesions were encountered, and adhesiolysis was performed. A 12 x 10 cm left lower quadrant tumor mass involved the sigmoid colon, urinary bladder, and two small-bowel loops. En bloc resection was carried out, including sigmoid colectomy, partial cystectomy (closed in two layers), and two small-bowel resections with primary anastomoses (two small bowel-to-small bowel anastomoses and a stapled colonic anastomosis). A second tumor mass in the left upper quadrant, measuring approximately 10 x 9 cm, involved the omentum and splenocolic ligament and was removed en bloc with splenectomy. Additional small nodules were excised, and a 5 x 4 cm abdominal wall lesion involving the right rectus abdominis muscle was resected with the involved muscle (Figure 2).

**Figure 3:**
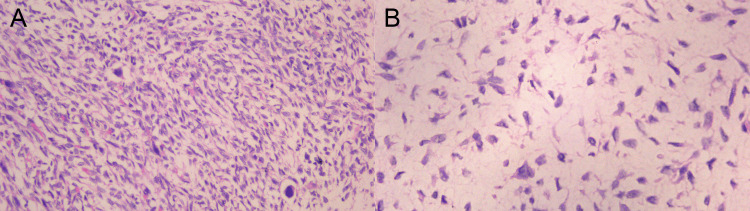
Representative hematoxylin and eosin (H&E) sections from the 2025 resection Panel A (20x) shows a spindle-cell neoplasm with variable cellularity in a collagenous to focally myxoid stroma. Panel B (40x) highlights bland elongated spindle cells with minimal cytologic atypia.

Histopathology from the 2025 resection was consistent with recurrent LGFMS (Figure 3), and surgical margins were negative. The postoperative course was uncomplicated. The patient was transferred from the intensive care unit to the surgical ward on postoperative day 4 and recovered without complications. Postoperative surveillance was performed with whole-body contrast-enhanced CT (chest/abdomen/pelvis) at the patient’s follow-up oncology center. The most recent scan, obtained six months after the July 2025 resection, showed no evidence of recurrent or metastatic disease.

## Discussion

LGFMS is clinically important because histologic blandness does not reliably predict behavior. Long-term series and reviews document meaningful risks of late recurrence and metastasis [1,2]. Intra-abdominal disease can amplify these challenges: tumors may grow with minimal symptoms, recur without early clinical signs, and become technically difficult to clear due to proximity to bowel, mesentery, and other critical viscera [2]. Our patient illustrates this pattern. Despite an initially margin-negative resection, she developed repeated intra-abdominal recurrences over four years, requiring escalating surgical strategies to achieve macroscopic clearance while maintaining oncologic principles.

For resectable disease, complete excision with negative margins is the key surgical determinant of local control [8,9]. In the reoperative abdomen, adhesions and distorted tissue planes increase the risk of tumor violation and incomplete excision. An en bloc approach is therefore often required when the tumor is adherent to or involves adjacent organs. In our case, operative management prioritized a no-touch technique and organ resection when necessary to avoid fragmentation and preserve oncologic planes. In 2025, multifocal disease involving the sigmoid colon, urinary bladder, and two small-bowel loops required multivisceral en bloc resection, and synchronous upper-quadrant involvement necessitated splenectomy as part of complete excision. Negative margins were achieved, supporting the feasibility of margin-oriented surgery even in complex, reoperative intra-abdominal settings.

Accurate pathologic classification is essential because management hinges on resectability and margin status. When molecular testing is unavailable, diagnosis commonly relies on morphology supported by immunohistochemistry. MUC4 is a highly sensitive marker for LGFMS and is valuable in the differential diagnosis of intra-abdominal myxoid/spindle-cell tumors [3]. Molecularly, LGFMS is strongly associated with FUS/CREB3L2 and, less commonly, with FUS/CREB3L1; these findings support diagnosis when available [4,5]. A practical pitfall is the recognized relationship within the LGFMS-SEF spectrum and the presence of overlapping features in some tumors. MUC4 is also useful in SEF and can support recognition of related entities when interpreted in context [6,7]. In our patient, consistent histopathologic interpretation across multiple resections favored recurrence rather than a new primary process.

The role of systemic therapy in LGFMS is uncertain and frequently limited. Retrospective outcome data in advanced LGFMS and related entities suggest that chemotherapy and other systemic regimens most often provide disease stabilization in selected cases rather than durable control [10,11]. In general soft-tissue sarcoma practice, anthracycline-based regimens (such as doxorubicin, with or without ifosfamide) are commonly used first-line for advanced, unresectable disease, while histology-specific responses vary and the benefit in LGFMS is inconsistent [9-11]. In our patient, systemic therapy was administered as part of oncologic management at an external center, but regimen details were not available in our records. Repeated recurrence in this case reinforces the practical importance of a surgery-first approach when complete resection is feasible.

Given the protracted natural history of LGFMS, surveillance should be ongoing and structured, with attention to both local relapse (which may be clinically silent) and distant disease [2,9]. In our case, the 2024 recurrence was detected at approximately 7 cm, underscoring that intra-abdominal relapse may be substantial by the time it is identified. This supports close, scheduled cross-sectional imaging follow-up after resection. Our patient is disease-free at six months after the most recent resection, which is encouraging but represents short follow-up relative to the known behavior of LGFMS.

## Conclusions

Recurrent intra-abdominal LGFMS can follow a protracted course with repeated relapse despite an indolent histologic appearance. In this case, complete surgical excision with negative margins achieved disease control across multiple operations. The final procedure required multivisceral en bloc resection for multifocal involvement. For resectable recurrent disease, surgery should remain the primary treatment strategy, coupled with ongoing surveillance, given the potential for late recurrence and metastasis.
